# Effect of *Bacillus* Probiotics on Growth Performance, Diarrhea Incidence, Nutrient Digestibility, and Intestinal Health of Weaned Piglets

**DOI:** 10.3390/ani15243629

**Published:** 2025-12-17

**Authors:** Xinhong Wang, Siqi Liu, Zihan Zhu, Chunyan Guo, Yinghai Jin, Zhenlong Wu, Xianren Jiang

**Affiliations:** 1 Key Laboratory of Feed Biotechnology of Ministry of Agriculture and Rural Affairs, Institute of Feed Research, Chinese Academy of Agriculture Sciences, Beijing 100081, China; wangxinhong1010@163.com; 2 College of Agriculture, Yanbian University, Yanji 133002, China; jinyh@ybu.edu.cn; 3 College of Animal Science and Technology, China Agricultural University, Beijing 100193, China; siqi_liu647@126.com; 4 Beijing Agricultural Science Animal Nutrition Research Center, Beijing 100021, China; zhuzihan0606@163.com; 5 Novonesis, Beijing 100085, China; chgu@novonesis.com

**Keywords:** *Bacillus*, diarrhea rate, intestinal barrier function, weaned piglets

## Abstract

Weaned piglets have a fragile gastrointestinal tract and immature digestive function. Supplementation of Bacillus can enhance intestinal barrier function and improve nutrient digestion and absorption efficiency. It is an important nutritional regulation method to alleviate weaning stress, reduce the incidence of diarrhea and promote growth performance. This experiment was conducted to investigate the effects of *Bacillus subtilis*, *Bacillus pumilus* and their combination on growth performance, diarrhea incidence, nutrient apparent digestibility, intestinal morphology and barrier function of weaned piglets. A total of 128 weaned piglets weighing 6.68 kg (±0.35 kg) were selected, divided into 4 treatment groups, and fed with a basal diet (CTR), a *Bacillus subtilis* (BS1), a *Bacillus pumilus* (BS2) and a *Bacillus subtilis* + *Bacillus pumilus* (BS1 + BS2) for 42 days. Each group had 8 replicates with 4 piglets per replicate. One piglet was selected from each replicate and euthanized to collect intestinal samples. The results showed that compared with the CTR group, the BS1 + BS2 group significantly increased the average daily gain (ADG) of weaned piglets on days 0–14 (*p* < 0.05), and the BS2 group significantly increased the ADG on days 0–42 (*p* < 0.05). Compared with the CTR group, the BS1 + BS2 group significantly reduced the full-time diarrhea rate (*p* < 0.05), and weaned piglets of the BS2 group significantly reduced the incidence of diarrhea on days 0–14, 15–28, and 0–42 of the study, in comparison to the control (*p* < 0.05). Compared with the CTR group, the piglets in the BS1 + BS2 group significantly decreased the serum tumor necrosis factor-α (TNF-α) content on day 21 (*p* < 0.05), and the BS1, BS2, and BS1 + BS2 groups significantly decreased the serum TNF-α content on day 42 (*p* < 0.05). Compared with the CTR group, the BS1 + BS2 group significantly reduced the expression of Interleukin-8 (*IL-8*) mRNA in the ileum (*p* < 0.05). The BS1, BS2, and BS1 + BS2 groups significantly reduced the expression of *TNF-α* mRNA in the ileum, *IL-8* mRNA in the jejunum, and *TNF-α* mRNA in the jejunum (*p* < 0.05). In addition, compared with the CTR group, the BS2 and BS1 + BS2 groups significantly increased *Claudin-1* mRNA expression in the jejunum (*p* < 0.05). Compared with the CTR group, the BS1 and BS2 groups significantly increased *Occludin* mRNA expression in the jejunum (*p* < 0.05). In summary, dietary supplementation with *Bacillus*-based probiotics can significantly improve growth performance in weaned piglets, reduce diarrhea incidence, alleviate inflammation, and enhance intestinal barrier function.

## Simple Summary

Whether the piglets can successfully pass the post-weaning stage directly determines the production efficiency of the farm. Bacillus has shown great potential in the field of animal nutrition because of its important roles in antioxidant capacity, anti-inflammatory effect and immune regulation. This study shows that the addition of *Bacillus* probiotics can improve the growth performance of weaned piglets, reduce the diarrhea rate, improve the antioxidant and anti-inflammatory ability, and maintain intestinal health.

## 1. Introduction

With the continued development of intensive breeding, early weaning has become an essential step in pig breeding in recent years. After weaning, piglets experience a series of weaning stressors due to incomplete gastrointestinal development, an immature immune system, and weak disease resistance, as well as the influence of diet composition, feeding environment, and psychological factors, which lead to developmental delay, diarrhea, and even death [1,2]. In the past, antibiotics were added to the diet to alleviate early weaning stress in piglets. However, the addition of antibiotics to the diet could lead to bacterial resistance and result in drug residues in animals [3,4]. The European Union (EU) banned the use of antibiotic growth promoters in animal feed in 2006, which posed a significant challenge for ensuring the healthy growth of weaned piglets. Therefore, any reliable strategy that enhances the anti-stress ability of weaned piglets offers tremendous benefits to the industry [5,6].

At present, it is generally believed that probiotics are living microorganisms. When ingested in sufficient quantities, substances that bring health benefits to the host [7]. Typically, three kinds of probiotics can be fed directly and are beneficial to the host: Yeast, Lactic acid bacteria, and *Bacillus* [5,8]. More and more studies have shown that adding probiotics to the diet can regulate the intestinal flora and confer numerous health benefits to weaned piglets. Its benefits include improving growth performance and nutrient digestibility, inhibiting pathogen growth, and enhancing immunity [9,10,11]. Compared with other types of probiotics, *Bacillus*-based probiotics have clear advantages because they can form a thick, hydrophobic spore shell, which effectively enhances their resistance to harsh gastrointestinal environments, thereby laying a foundation for successful colonization in the intestine [12,13]. Up to now, many studies have shown that *Bacillus subtilis* supplementation could improve the intestinal health of pigs by changing the intestinal barrier function, thereby inhibiting the growth of pathogens, enhancing immune function, improving nutrient utilization and digestibility, reducing the incidence of diarrhea, and ultimately improving the growth performance of piglets [14,15,16]. However, there are few studies on the effects of adding *Bacillus pumilus* to weaned piglets. Therefore, this experiment was conducted to investigate the effects of *Bacillus subtilis*, *Bacillus pumilus*, and their combination on growth performance, diarrhea rate, nutrient apparent digestibility, intestinal morphology, and intestinal barrier function of weaned piglets, and to provide data reference for the in-depth research and development of *Bacillus* probiotics in animal production.

## 2. Materials and Methods

### 2.1. Animal Ethics Approval

The animal procedures in this study were approved by the Animal Care and Use Committee of the Institute of Feed Research of the Chinese Academy of Agricultural Sciences (IFR-CAAS20240515, 15 May 2024). This experiment was carried out in the experimental pig farm of Tianpeng Animal Husbandry Co., Ltd., Langfang City, Hebei Province.

### 2.2. Animals, Feeding, Experimental Designs and Sample Collection

A total of 128 weaned piglets (Duroc × Landrace × Yorkshire) with an average initial body weight (BW, 6.68 kg ± 0.35) and age (21 ± 1 days) were used. Weaned piglets were randomly assigned to four treatments with eight replicate pens per treatment and four piglets per pen. The experiment lasted 42 days, divided into two stages: the early nursery phase (days 0–14) and the late nursery phase (days 14–42). The diets of the four treatment groups included: control group (CTR): fed a basal diet; three groups (BS1, BS2, and BS1 + BS2) fed a basal diet supplemented with 0.05% *Bacillus subtilis*, 0.05% *Bacillus pumilus*, and 0.05% *Bacillus subtilis* + 0.05% *Bacillus pumilus*. Novonesis, Kongens Lyngby, Denmark, provided the two Bacillus strains used in this trial. The viable count of both probiotics was 5.4 × 10^8^ CFU/g and the carriers were calcium carbonate. In addition, the strain ID of *Bacillus subtilis* was O7SKS, while the strain ID of *Bacillus pumilus* was O72NR7. The corn-soybean meal basal diet was formulated to meet the nutritional requirements of the National Research Council (NRC) [8] and did not contain any antibiotic growth promoters, as shown in Table 1. The temperature in the nursery house was controlled at 26–28 °C, and the relative humidity was maintained at 55–65%. Piglets were given ad libitum access to feed and fresh water through a feed trough and nipples in pens with slatted floors.

One piglet was randomly selected from each pen, and feces were collected on days 21 and days 40, 41, and 42 for subsequent analysis of indices. On days 21 and 42, blood samples were collected from the anterior vena cava. A total of 8 mL of blood was collected from each piglet into a vacuum tube, and then centrifuged at 3000 r/min at 4 °C for 10 min to obtain serum. The serum was stored at −20 °C for analysis of antioxidant capacity, inflammatory factors, and immunoglobulins.

On day 42 of the trial, a piglet with an average body weight (BW) was selected from each replicate. The piglet was stunned in a 100 cm × 65 cm × 54 cm uncovered plastic box using a portable electric shocker (output voltage 220 V), and the piglet was bled quickly to euthanize it. The abdomen was then longitudinally incised to collect the target tissues. About 15 cm of tissue was harvested from the proximal ileum and jejunum. The first intestinal segment, approximately 4 cm, was fixed in fresh 4% paraformaldehyde for 24 h and then stored in 70% ethanol for microscopic evaluation of jejunum morphology (including villus height (VH), crypt depth (CD), and villus height to crypt depth ratio (V:C)). The remaining sections were cut longitudinally to expose the mucosa and washed three times with phosphate-buffered saline to remove mucus and digesta. Then, the mucosa was gently scraped off with a glass microscope slide, placed in a low-temperature cryopreservation tube, quickly frozen in liquid nitrogen, and subsequently used for detection of mucosal antioxidants was performed.

**Table 1:** Ingredient composition of the diets (%, as-fed basis).

	0–14 Day				14–42 Day			
	CTR	BS1	BS2	BS1 + BS2	CTR	BS1	BS2	BS1 + BS2
Ingredients, %								
Corn	46.00	46.00	46.00	46.00	60.32	60.32	60.32	60.32
Soybean meal, 43%	16.20	16.20	16.20	16.20	18.50	18.50	18.50	18.50
Expanded soybean	12.90	12.90	12.90	12.90	7.5	7.5	7.5	7.5
Fish meal, 65%	6.00	6.00	6.00	6.00	4.00	4.00	4.00	4.00
Whey powder	14.80	14.80	14.80	14.80	5.00	5.00	5.00	5.00
Soybean oil	1.00	1.00	1.00	1.00	1.00	1.00	1.00	1.00
Calcium dihydrogen phosphate	0.35	0.35	0.35	0.35	0.60	0.60	0.60	0.60
Limestone	0.77	0.77	0.77	0.77	1.00	1.00	1.00	1.00
Salt	0.40	0.40	0.40	0.40	0.40	0.40	0.40	0.40
L-Lysine HCL, 55%	0.38	0.38	0.38	0.38	0.53	0.53	0.53	0.53
DL-Methionine	0.03	0.03	0.03	0.03	0.05	0.05	0.05	0.05
Threonine	0.08	0.08	0.08	0.08	0.11	0.11	0.11	0.11
Tryptophan	0	0	0	0	0.01	0.01	0.01	0.01
Bran	0.552	0.502	0.502	0.452	0.642	0.592	0.592	0.592
Choline chloride, 60%	0.05	0.05	0.05	0.05	0.05	0.05	0.05	0.05
Phytase (10,000) ^1^	0.02	0.02	0.02	0.02	0.02	0.02	0.02	0.02
Premix ^2^	0.268	0.268	0.268	0.268	0.088	0.088	0.088	0.088
Zinc oxide	0.20	0.20	0.20	0.20	0	0	0	0
*Bacillus subtilis*	0	0.05	0	0.05	0	0.05	0	0.05
*Bacillus pumilus*	0	0	0.05	0.05	0	0	0.05	0.05
Nutrition composition								
Analyzed value								
Crude protein	20.37	20.33	20.46	20.36	19.29	19.34	19.55	19.36
Calcium	0.83	0.79	0.85	0.88	0.73	0.71	0.69	0.75
Total phosphorus	0.64	0.60	0.62	0.59	0.56	0.53	0.57	0.54
Ether extract	5.21	5.39	5.33	5.34	3.96	4.17	4.07	4.27
Crude Ash	5.77	5.82	5.83	5.69	4.92	4.70	4.88	4.83
Calculated value								
Metabolizable energy, kcal/kg	3400	3400	3400	3400	3350	3350	3350	3350
SID Lysine	1.3	1.3	1.3	1.3	1.15	1.15	1.15	1.15
SID Methionine	0.38	0.38	0.38	0.38	0.36	0.36	0.36	0.36
SID Threonine	0.76	0.76	0.76	0.76	0.68	0.68	0.68	0.68
SID Tryptophan	0.21	0.21	0.21	0.21	0.19	0.19	0.19	0.19
SID Valine	0.76	0.76	0.76	0.76	0.70	0.70	0.70	0.70
SID Isoleucine	0.71	0.71	0.71	0.71	0.64	0.64	0.64	0.64

SID = Standardized ileal digestibility. ^1^ 1 × 10^4^ phytase unit per kg of products (VTR Biotechnology Co., Ltd., Zhuhai, China). ^2^ Premix supplied per kg of diet: vitamin A, 35.2 mg; vitamin D3, 7.68 mg; DL-α-tocopherol, 128 mg; menadione sodium bisulfite, 8.16 mg; thiamine mononitrate, 4 mg; riboflavin, 12 mg; pyridoxine hydrochloride, 8.32 mg; cyanocobalamin, 4.8 mg; niacin, 38.4 mg; calcium pantothenate, 25 mg; folic acid, 1.68 mg; biotin, 0.16 mg; iron (FeSO_4_·H_2_O), 171 mg; manganese (MnSO_4_·H_2_O), 42.31 mg; copper (CuSO_4_·5H_2_O), 125 mg; selenium (Na_2_SeO_3_), 0.19 mg; cobalt (CoCl_2_), 0.19 mg; iodine (Ca(IO_3_)_2_), 0.54 mg.

### 2.3. Growth Performance and Incidence of Diarrhea

Body weight and feed intake were recorded on days 0, 14, 28, and 42 for each pen to assess average daily gain (ADG), average daily feed intake (ADFI), and feed conversion ratio (FCR). According to the 5-point fecal consistency scoring system, the diarrhea score was recorded by the same person at 9:00 every day: 1 = hard, dry pellet; 2 = firm, formed stool; 3 = soft, moist stool that maintains its shape; 4 = soft, shapeless feces; and 5 = flowable liquid. Liquid form of feces (4–5 points) is considered diarrhea. Diarrhea rate (%) = [total number of diarrhea in each group/(experiment days × number of piglets in each group)] × 100 [17].

### 2.4. Apparent Digestibility of Nutrients

Apparent total tract digestibility (ATTD) was determined by the endogenous indicator method. Acid-insoluble ash (AIA) was used as an endogenous indicator to analyze moisture (method 930.15) [18] and crude protein (N × 6.25) (Methods 990.03) in diet and fecal samples [18]. Gross energy (GE) was measured using a Parr 6400 calorimeter (Parr Instrument Company, Moline, IL, USA). The apparent digestibility of nutrients was calculated using AIA as an internal marker. The AIA content in the diet and feces was determined according to the method described by Newkirk et al. [19].

### 2.5. Serum Antioxidant Indexes and Oxidative Stress Biomarker

Thawed serum was evaluated for Superoxide dismutase (SOD, U/mL), malondialdehyde (MDA, nmol/mL), and glutathione peroxidase (GSH-Px, U/mL) in plasma, which were measured by commercial kits (Jiancheng Bioengineering Institute (Nanjing) Co., Ltd., Nanjing, China), and the operation steps were strictly in accordance with the instructions. The activity of SOD was determined by the WST-1 method, and the absorbance was measured at 450 nm. The level of MDA was determined by the thiobarbituric acid method, and the absorbance was measured at 532 nm. The activity of GSH-Px was determined by the dithiodinitrobenzoic acid method, and the absorbance was measured at 412 nm.

### 2.6. Antioxidant Indexes of Jejunum Mucosa

The protein concentration of the jejunal mucosa was determined by a commercial kit (Huaxing Biotechnology (Beijing) Co., Ltd., Beijing, China). About 50 mg of jejunal mucosa powder was added to 0.2 mL of 0.9% normal saline, homogenized, and fully homogenized. The supernatant was diluted and mixed 10 times, then 20 μL was transferred to 96-well plates, 200 μL of WR working solution was added, incubated at 37 °C for 30 min, and the supernatant was collected for detection. According to the manufacturer’s instructions, the levels of total superoxide dismutase (T-SOD, U/mL), catalase (CAT, U/mL), 8-hydroxydeoxyguanosine (8-OHdG, ng/mL), and total antioxidant capacity (T-AOC, U/mL) in jejunal mucosa were determined using commercial detection kits (Jiancheng Bioengineering Institute (Nanjing) Co., Ltd.).

### 2.7. Serum Inflammation and Immune Indexes (pg/mL)

The inflammatory markers of serum samples were further analyzed, including interleukin-6 (IL-6), interleukin-1β (IL-1β), tumor necrosis factor-α (TNF-α) and interferon-γ (IFN-γ). The serum immune indexes, including immunoglobulin A (IgA), immunoglobulin G (IgG) and immunoglobulin M (IgM) were measured. The test steps of the above kits follow the instructions (Jiancheng Biotengineering Institute (Nanjing) Co., Ltd.).

### 2.8. Intestinal Morphology

The jejunum and ileum specimens were dehydrated using a graded ethanol series, rinsed with xylene, and embedded in paraffin. Then, 10 sections, each 5 μm thick were stained with hematoxylin and eosin. Six intact villi and crypt structures were observed under a microscope, and VH and CD measurements were performed using Image-Pro Plus 6.0 (Media Cybernetics, Singapore).

### 2.9. Real-Time Quantitative PCR

RNA extraction was performed using a commercial kit (Apollo Scientific Instruments (Jiangsu) Co., Ltd., Nantong, China) according to the manufacturer’s instructions. RNA was obtained, and its concentration and quality were determined using a NanoDrop (Thermo Fisher Scientific, Waltham, MA, USA) to ensure that the A260/280 and A260/230 ratios were between 1.8 and 2.1 and 2.0 and 2.5, respectively. The sample concentration was then adjusted to about 1000 ng/μL using RNase-free Water for subsequent reverse transcription. The commercial kit (Takara Biomedical Technology (Beijing) Co., Ltd., Beijing, China) was used for reverse transcription in accordance with the instructions’ operating steps. The reverse transcription system was 20 μL, and the RNA volume required for reaction was calculated according to the above RNA concentration. 4 μL (5× UltraScript RT MasterMix) and 1 μL (gDNA Remover) were added, respectively. The RNase-free Water system was used for reverse transcription using a PCR instrument (Bio-Rad, Hercules, CA, USA) to obtain the cDNA from the sample. After proper dilution and mixing, it was stored at −20 °C for testing. Fluorescent dyes were purchased from the company (Takara Biomedical Technology (Beijing) Co., Ltd.), and the primer sequences were sent to the company for synthesis (Tianyi Huiyuan (Beijing) Co., Ltd., Beijing, China). The primer sequence information is shown in Table 2. The reaction system was 20 μL, including 2 μL sample cDNA, 0.5 μL forward primer, 0.5 μL reverse primer, 10 μL TB Green, and 7 μL RNase-free Water. GAPDH was used as an internal reference gene, and the CFX96 real-time PCR instrument (Bio-Rad, USA) was used for real-time fluorescence quantitative analysis. The relative expression of the target gene was calculated using the 2^−ΔΔCT^ method.

**Table 2:** Primer sequences for real-time fluorescent quantitative PCR.

Gene	Accession NO.	Primer Sequences (5′-3′)	Product Length, bp
GAPDH	NM_001206359.1	F: GCTTGTCATCAATGGAAAGG	86
		R: CATACGTAGCACCAGCATCA	
*IL-6*	NM_214399.1	F: ACAAAGCCACCACCCCTAAC	82
		R: CGTGGACGGCATCAATCTCA	
*IL-8*	NM_213867.1	F: CCGTGTCAACATGACTTCCAA	75
		R: GCCTCACAGAGAGCTGCAGAA	
*IL-10*	NM_214041.1	F: GACGATGAAGATGAGGAAGA	54
		R: AGGTTTTTCTTTGGTTTCCC	
*TNF-α*	NM_214022.1	F: CTCACGTCCTTCTGGTTTAG	96
		R: CCCTGATTTCTAAGTGTTGC	
*Claudin-1*	NM_001244539.1	F: CCTCAATACAGGAGGGAAGC	76
		R: CTCTCCCCACATTCGAGATGATT	
*Occludin*	NM_001163647.2	F: TCAGGTGCACCCTCCAGATT	112
		R: TGGACTTTCAAGAGGCCTGG	
*ZO-1*	CV870309	F: CGATCACTCCAGCATACAAT	111
		R: CACTTGGCAGAAGATTGTGA	

*IL-6*, interleukin-6; *IL-8*, interleukin-8; *IL-10*, interleukin-10; *TNF-α*, tumor necrosis factor-α; *ZO-1*, zonula occludens-1.

### 2.10. Statistical Analysis

SAS 9.4 (SAS Institute, 2009, Cary, NC, USA) was used to analyze the growth performance data in the experiment by block analysis, the diarrhea rate data in the experiment were analyzed by the chi-square test, the remaining data in the experiment were analyzed by single-factor ANOVA, and Tukey was used for post hoc multiple comparison. Differences were considered statistically significant at *p* < 0.05. When 0.05 < *p* ≤ 0.10, the trend was considered significant.

## 3. Results

### 3.1. Growth Performance of Weaned Piglets

Table 3 shows the effect of *Bacillus* probiotics on the growth performance of weaned piglets. Compared with the CTR group, the BS1 + BS2 group showed a significant increase in BW on day 14 (*p* < 0.05). At the same time, the BS1, BS2 and BS1 + BS2 groups significantly increased the BW on days 28 and 42 (*p* < 0.05), compared with the BS2 and BS1 + BS2 groups, the BS1 group significantly increased the BW on day 28 (*p* < 0.05). Compared with the CTR and BS1 groups, the BS1 + BS2 group significantly increased ADG on days 0–14 (*p* < 0.05); compared with the CTR and BS1 + BS2 groups, the BS1 and BS2 groups significantly increased ADG on days 14–28 (*p* < 0.05); compared with the CTR group, the BS1, BS2 and BS1 + BS2 groups significantly increased ADG on days 28–42 (*p* < 0.05); compared with the BS2 and BS1 + BS2 groups, the BS1 group significantly increased ADG on days 28–42 (*p* < 0.05); compared with the CTR group, the BS1, BS2 and BS1 + BS2 groups significantly increased ADG on days 0–42 (*p* < 0.05). Compared with the CTR, BS2 and BS1 + BS2 groups, the ADFI of the BS1 group was significantly increased on days 14–28 and 28–42 (*p* < 0.05); compared with the BS1 + BS2 group, the BS2 group significantly increased ADFI on days 14–28 and 28–42 (*p* < 0.05); compared with the CTR, BS1 and BS1 + BS2 groups, the BS2 group significantly increased ADFI on days 0–42 (*p* < 0.05). Compared with the CTR group, the BS1, BS2 and BS1 + BS2 groups significantly reduced FCR on days 0–14 and 14–28 (*p* < 0.05), and compared with the BS1 + BS2 group, the BS2 group significantly reduced FCR on days 14–28 (*p* < 0.05); compared with the CTR and BS2 groups, the BS1 and BS1 + BS2 groups significantly reduced FCR on days 28–42 (*p* < 0.05); compared with the CTR group, the BS1, BS2 and BS1 + BS2 groups significantly reduced FCR on days 0–42 (*p* < 0.05), and compared with the BS2 group, the BS1 + BS2 group significantly reduced FCR on days 0–42 (*p* < 0.05).

**Table 3:** Effects of *Bacillus* probiotics on growth performance of weaned piglets ^1^.

	CTR	BS1	BS2	BS1 + BS2	SEM	*p*-Value
BW, kg						
Day 0	6.76	6.85	6.76	6.71	0.10	0.769
Day 14	11.08 ^b^	11.44 ^ab^	11.58 ^ab^	11.91 ^a^	0.19	0.025
Day 28	16.78 ^b^	17.85 ^a^	17.96 ^a^	17.81 ^a^	0.19	<0.001
Day 42	26.09 ^c^	29.19 ^a^	28.10 ^b^	28.06 ^b^	0.24	<0.001
ADG, g						
Day 0–14	308 ^b^	328 ^b^	344 ^ab^	371 ^a^	11	<0.001
Day 14–28	407.14 ^b^	457.52 ^a^	455.65 ^a^	421.88 ^b^	8.24	<0.001
Day 28–42	665 ^c^	810 ^a^	724 ^b^	732 ^b^	21	<0.001
Day 0–42	460.20 ^b^	531.81 ^a^	508.12 ^a^	508.44 ^a^	6.82	<0.001
ADFI, g						
Day 0–14	362	364	372	388	10.05	0.252
Day 14–28	624.69 ^bc^	653.91 ^a^	634.70 ^b^	612.44 ^c^	5.31	<0.001
Day 28–42	1189 ^bc^	1293 ^a^	1231 ^b^	1144 ^c^	17	<0.001
Day 0–42	725.28 ^b^	770.30 ^b^	745.97 ^a^	714.69 ^b^	6.69	<0.001
FCR						
Day 0–14	1.203 ^a^	1.118 ^b^	1.098 ^b^	1.057 ^b^	0.030	0.006
Day 14–28	1.552 ^a^	1.434 ^bc^	1.402 ^c^	1.478 ^b^	0.026	0.001
Day 28–42	1.808 ^a^	1.623 ^b^	1.791 ^a^	1.577 ^b^	0.052	0.003
Day 0–42	1.579 ^a^	1.455 ^bc^	1.477 ^b^	1.409 ^c^	0.017	<0.001

BW, body weight; ADG, average daily gain; ADFI, average daily feed intake; FCR, feed conversion ratio; CTR, base diet without additives; BS1, CTR + 0.05% *Bacillus subtilis*; BS2, CTR + 0.05% *Bacillus pumilus*; BS1 + BS2, CTR + 0.05% *Bacillus subtilis* + 0.05% *Bacillus pumilus*. ^1^ n = 8. ^a, b, c^ Means with different superscripts means significant difference (*p* < 0.05).

### 3.2. Diarrhea Incidence

Table 4 shows the effect of *Bacillus* probiotics on the diarrhea rate of weaned piglets. Compared with the CTR group, the BS1 + BS2 group significantly reduced the diarrhea rate on days 0–14,14–28, and 28–42 (*p* < 0.05); compared with the CTR group, the BS1 and BS2 groups significantly reduced the diarrhea rate on days 14–28 (*p* < 0.05); compared with the CTR group, the BS2 group significantly reduced the diarrhea rate on days 28–42 (*p* < 0.05); compared with the CTR group, the BS1, BS2 and BS1 + BS2 groups significantly reduced the diarrhea rate on days 0–42 (*p* < 0.05).

**Table 4:** Effect of *Bacillus* probiotics in feed on diarrhea incidence of weaned piglets (%) ^1^.

	CTR	BS1	BS2	BS1 + BS2	SEM	*p*-Value
Day 0–14	12.39 ^a^	10.13 ^abc^	10.83 ^ab^	7.02 ^c^	-	0.046
Day 14–28	8.26 ^a^	3.76 ^b^	3.38 ^b^	4.68 ^b^	-	0.006
Day 28–42	2.15 ^a^	0.80 ^ab^	0.26 ^b^	0.54 ^b^	-	0.041
Day 0–42	8.34 ^a^	5.25 ^b^	5.17 ^b^	4.33 ^b^	-	<0.001

CTR, base diet without additives; BS1, CTR + 0.05% *Bacillus subtilis*; BS2, CTR + 0.05% *Bacillus pumilus*; BS1 + BS2, CTR + 0.05% *Bacillus subtilis* + 0.05% *Bacillus pumilus*. ^1^ n = 8. ^a, b, c^ Means with different superscripts means significant difference (*p* < 0.05).

### 3.3. Nutrient Apparent Digestibility

Table 5 shows the effect of *Bacillus* probiotics on the nutrient apparent digestibility of weaned piglets. Compared with the CTR group, the dry matter (DM) digestibility of weaning piglets in BS1, BS2, and BS1 + BS2 groups was significantly increased on days 14 and 42 (*p* < 0.05), and compared with the BS1 and BS2 groups, the DM digestibility of the BS1 + BS2 group was significantly increased on day 21 (*p* < 0.05). In addition, compared with the CTR group, the BS2 and BS1 + BS2 groups significantly increased the digestibility of crude protein (CP) and gross energy (GE) on day 42 (*p* < 0.05). There was no significant difference in the digestibility of ash content (ASH), calcium (Ca), and ether extract (EE) among the groups (*p* > 0.05).

**Table 5:** Effects of dietary *Bacillus* probiotics on nutrient apparent digestibility of weaned piglets (%, as-DM basis) ^1^.

	CTR	BS1	BS2	BS1 + BS2	SEM	*p*-Value
DM						
Day 21	69.49 ^c^	72.31 ^b^	73.10 ^b^	76.43 ^a^	0.67	<0.001
Day 42	74.27 ^b^	78.22 ^a^	78.39 ^a^	78.89 ^a^	0.93	0.009
CP						
Day 21	67.26	68.27	68.79	70.63	2.00	0.701
Day 42	70.90 ^b^	74.35 ^ab^	79.26 ^a^	78.90 ^a^	1.05	0.004
ASH						
Day 21	42.84	47.87	48.89	49.20	1.88	0.648
Day 42	35.45	37.01	38.77	43.72	1.65	0.441
Ca						
Day 21	61.79	62.90	64.34	64.97	2.00	0.948
Day 42	61.40	63.54	63.31	70.17	3.66	0.104
P						
Day 21	60.65	67.39	67.66	68.73	2.78	0.212
Day 42	65.94 ^y^	67.22	66.54 ^y^	72.93 ^x^	1.06	0.075
EE						
Day 21	66.74	70.41	69.55	72.91	1.07	0.277
Day 42	59.28 ^b^	66.26 ^a^	66.67 ^a^	67.19 ^a^	1.14	0.044
GE						
Day 21	76.76	77.66	77.52	78.97	0.93	0.884
Day 42	79.98 ^b^	83.90 ^a^	85.46 ^a^	85.08 ^a^	0.71	0.011

DM, dry matter; CP, crude protein; ASH, ash content; Ca, calcium; P, phosphorus; EE, ether extract; GE, gross energy. CTR, base diet without additives; BS1, CTR + 0.05% *Bacillus subtilis*; BS2, CTR + 0.05% *Bacillus pumilus*; BS1 + BS2, CTR + 0.05% *Bacillus subtilis* + 0.05% *Bacillus pumilus* ^1^ n = 8. ^a, b, c^ Means with different superscripts means significant difference (*p* < 0.05). ^x, y^ Means listed in the same row with different superscripts tend to be different (0.05 < *p* ≤ 0.10).

### 3.4. Serum Antioxidant Capacity

Table 6 shows the effect of dietary *Bacillus* probiotics on the serum antioxidant capacity of weaned piglets. Compared with the CTR group, the BS1 + BS2 group had a higher trend of serum SOD activity on day 21 (*p* = 0.08) and the BS2 and BS1 + BS2 groups had a lower trend of MDA level (*p* = 0.054). In addition, compared with the CTR group, the BS2 and BS1 + BS2 groups significantly increased SOD activity (*p* < 0.05) and significantly decreased serum MDA level on day 42 (*p* < 0.05).

**Table 6:** Effects of dietary *Bacillus* probiotics on serum antioxidant capacity of weaned piglets ^1^.

	CTR	BS1	BS2	BS1 + BS2	SEM	*p*-Value
SOD, U/mL						
Day 21	16.3 ^y^	18.60	19.05	25.2 ^x^	1.30	0.080
Day 42	22.4 ^b^	25.56 ^ab^	30.14 ^a^	26.74 ^ab^	1.03	0.035
MDA, nmol/mL						
Day 21	5.68 ^x^	4.46	3.88 ^y^	3.23 ^y^	0.35	0.054
Day 42	5.34 ^a^	4.03 ^b^	3.95 ^b^	3.61 ^b^	0.24	0.040
GSH-Px, U/mL						
Day 21	358.59	359.29	358.18	349.41	5.83	0.701
Day 42	543.53	535.15	555.88	549.26	8.10	0.406

SOD, superoxide dismutase; MDA, malondialdehyde; GSH-Px, glutathione peroxidase. CTR, base diet without additives; BS1, CTR + 0.05% *Bacillus subtilis*; BS2, CTR + 0.05% *Bacillus pumilus*; BS1 + BS2, CTR + 0.05% *Bacillus subtilis* + 0.05% *Bacillus pumilus*. ^1^ n = 8. ^a, b^ Means with different superscripts means significant difference (*p* < 0.05). ^x, y^ Means listed in the same row with different superscripts tend to be different (0.05 < *p* ≤ 0.10).

### 3.5. Antioxidant Capacity of Jejunal Mucosa

Table 7 shows the effect of dietary *Bacillus* probiotics on the antioxidant capacity of jejunal mucosa of weaned piglets. Compared with the CTR and BS1 groups, the BS2 group significantly increased T-SOD activity (*p* < 0.05), and compared with the CTR group, the BS1, BS2 and BS1 + BS2 groups significantly increased CAT activity (*p* < 0.05).

**Table 7:** Effects of dietary *Bacillus* probiotics on antioxidant capacity of jejunal mucosa in weaned piglets ^1^.

	CTR	BS1	BS2	BS1 + BS2	SEM	*p*-Value
T-SOD, U/mL	2.72 ^b^	2.72 ^b^	3.37 ^a^	2.98 ^ab^	0.16	0.025
CAT, U/mL	5.23 ^b^	11.46 ^a^	11.3 ^a^	11.33 ^a^	0.89	0.002
8-OHDG, ng/mL	82.43	75.75	71.92	77.16	5.72	0.601
T-AOC, U/mL	2.22	2.99	3.51	2.81	0.32	0.695

T-SOD, total superoxide dismutase; CAT, catalase; 8-OHDG, 8-hydroxydeoxyguanosine; T-AOC, total antioxidant capacity. CTR, base diet without additives; BS1, CTR + 0.05% *Bacillus subtilis*; BS2, CTR + 0.05% *Bacillus pumilus*; BS1 + BS2, CTR + 0.05% *Bacillus subtilis* + 0.05% *Bacillus pumilus*. ^1^ n = 8. ^a, b^ Means with different superscripts means a significant difference (*p* < 0.05).

### 3.6. Serum Inflammatory Factors

Table 8 shows the effect of *Bacillus* probiotics on the content of serum inflammatory factors in weaned piglets. Compared with the CTR group, the BS1 + BS2 group had significantly decreased TNF-α level on day 21 (*p* < 0.05); compared with the CTR group, the BS1, BS2 and BS1 + BS2 groups had a significantly decreased TNF-α level on day 42 (*p* < 0.05); there was no significant difference in serum IFN-γ content between the groups (*p* > 0.05).

**Table 8:** Effects of dietary *Bacillus* probiotics on serum inflammatory factors in weaned piglets (pg/mL) ^1^.

	CTR	BS1	BS2	BS1 + BS2	SEM	*p*-Value
IL-1β						
Day 21	1245	1128 ^y^	1276 ^x^	1149	24	0.074
Day 42	1288	1207	1251	1217	35	0.365
TNF-α						
Day 21	316.50 ^a^	289.23 ^ab^	296.44 ^ab^	266.81 ^b^	9.90	0.013
Day 42	322 ^a^	262 ^b^	255 ^b^	257 ^b^	15	0.025
IL-6						
Day 21	1197	1123 ^y^	1214 ^x^	1145	20	0.054
Day 42	1109	1110	1065	1041	46	0.591
IFN-γ						
Day 21	54.67	54.85	58.46	58.57	2.25	0.427
Day 42	63.43	66.26	67.51	64.24	3.20	0.818

IL-1β, interleukin-1β; TNF-α, tumor necrosis factor-α; IL-6, interleukin-6; IFN-γ, interferon-γ. CTR, base diet without additives; BS1, CTR + 0.05% *Bacillus subtilis*; BS2, CTR + 0.05% *Bacillus pumilus*; BS1 + BS2, CTR + 0.05% *Bacillus subtilis* + 0.05% *Bacillus pumilus* ^1^ n = 8. ^a, b^ Means with different superscripts means a significant difference (*p* < 0.05). ^x, y^ Means listed in the same row with different superscripts tend to be different (0.05 < *p* ≤ 0.10).

### 3.7. Serum Immunity

Table 9 shows the effect of *Bacillus* probiotics on serum immune indexes of weaned piglets. There was no significant difference in serum immune indexes between the groups (*p* > 0.05).

**Table 9:** Effects of dietary *Bacillus* probiotics on serum immunity of weaned piglets (g/L) ^1^.

	CTR	BS1	BS2	BS1 + BS2	SEM	*p*-Value
IgA						
Day 21	11.02	9.65	10.40	10.55	4.98	0.301
Day 42	9.82	9.45	9.80	10.72	4.41	0.352
IgG						
Day 21	2.53	2.50	2.53	2.72	0.85	0.277
Day 42	3.10	2.96	3.17	3.10	1.99	0.912
IgM						
Day 21	2.69	2.43	2.89	2.53	1.57	0.215
Day 42	2.35	2.28	2.46	2.52	1.38	0.629

IgA, immunoglobulin A; IgG, immunoglobulin G; IgM, immunoglobulin M. CTR, base diet without additives; BS1, CTR + 0.05% *Bacillus subtilis*; BS2, CTR + 0.05% *Bacillus pumilus*; BS1 + BS2, CTR + 0.05% *Bacillus subtilis* + 0.05% *Bacillus pumilus* ^1^ n = 8.

### 3.8. Intestinal Morphology

**Figure 1:**
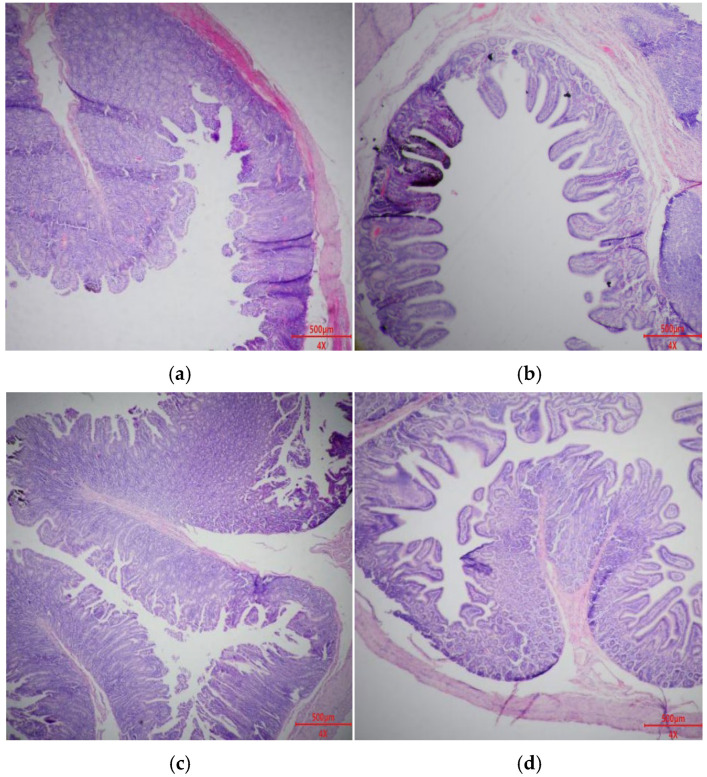
HE staining of ileum section (**a**) CTR, base diet without additives; (**b**) BS1, CTR + 0.05% *Bacillus subtilis*; (**c**) BS2, CTR + 0.05% *Bacillus pumilus*; (**d**) BS1 + BS2, CTR + 0.05% *Bacillus subtilis* + 0.05% *Bacillus pumilus*. n = 8.

**Figure 2:**
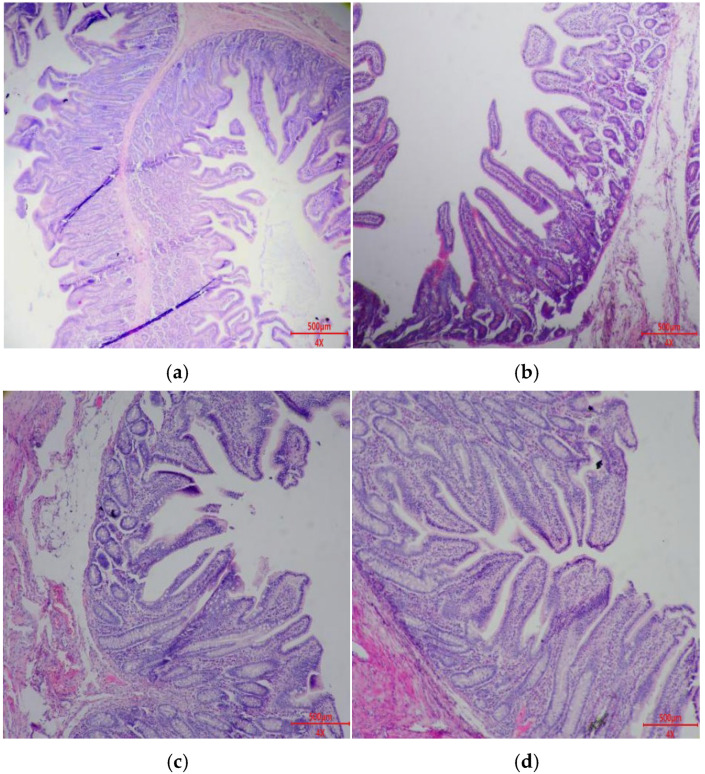
HE staining of jejunum section (**a**) CTR, base diet without additives; (**b**) BS1, CTR + 0.05% *Bacillus subtilis*; (**c**) BS2, CTR + 0.05% *Bacillus pumilus*; (**d**) BS1 + BS2, CTR + 0.05% *Bacillus subtilis* + 0.05% *Bacillus pumilus*. n = 8.

Figure 1 and Figure 2, and Table 10 show the effect of *Bacillus* probiotics on intestinal morphology of weaned piglets. Compared with the CTR and BS1 groups, the ileum CD and jejunum CD of the BS2 and BS1 + BS2 groups were significantly decreased (*p* < 0.05). In addition, compared with the CTR and BS1 groups, jejunum V:C in the BS2 and BS1 + BS2 groups was significantly increased (*p* < 0.05).

**Table 10:** Effects of dietary *Bacillus* probiotics on intestinal morphology of weaned piglets ^1^.

Items	CTR	BS1	BS2	BS1 + BS2	SEM	*p*-Value
Ileum						
VH, µm	388	461	392	380	14	0.141
CD, µm	272 ^a^	247 ^a^	175 ^b^	165 ^b^	12	0.008
V:C	1.44	1.92	2.27	2.33	0.144	0.079
Jejunum						
VH, µm	483	446	433	443	30	0.241
CD, µm	327 ^a^	265 ^a^	185 ^b^	181 ^b^	20	0.004
V:C	1.49 ^b^	1.71 ^b^	2.34 ^a^	2.48 ^a^	0.142	0.005

VH, including villus; CD, crypt depth; V:C, villus height to crypt depth ratio. CTR, base diet without additives; BS1, CTR + 0.05% *Bacillus subtilis*; BS2, CTR + 0.05% *Bacillus pumilus*; BS1 + BS2, CTR + 0.05% *Bacillus subtilis* + 0.05% *Bacillus pumilus*; ^1^ n = 8. ^a, b^ Means with different superscripts means a significant difference (*p* < 0.05).

### 3.9. mRNA Expression of Inflammatory Genes in Ileum Mucosa

**Figure 3:**
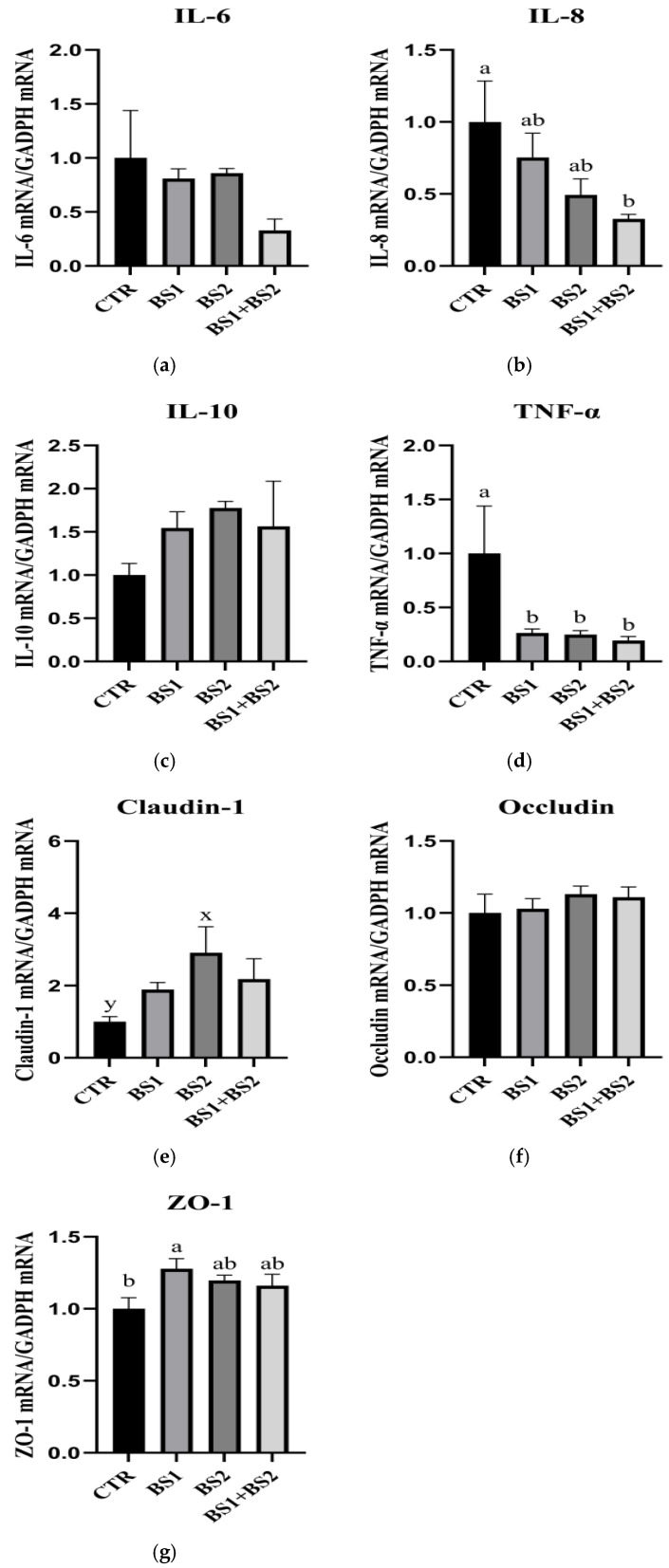
Effects of dietary *Bacillus* probiotics on mRNA expression of inflammatory genes in ileum mucosa of weaned piglets. *IL-6*, interleukin-6; *IL-8*, interleukin-8; *IL-10*, interleukin-10; *TNF-α*, tumor necrosis factor-α. CTR, base diet without additives; BS1, CTR + 0.05% *Bacillus subtilis*; BS2, CTR + 0.05% *Bacillus pumilus*; BS1 + BS2, CTR + 0.05% *Bacillus subtilis* + 0.05% *Bacillus pumilus*; (**a**) the expression of *IL-6* mRNA; (**b**) the expression of *IL-8* mRNA; (**c**) the expression of *IL-10* mRNA; (**d**) the expression of TNF-α; (**e**) the expression of Claudin-1 mRNA; (**f**) the expression of *Occludin* mRNA; (**g**) the expression of *ZO-1* mRNA. n = 8. ^a, b^ Means with different superscripts means significant difference (*p* < 0.05). ^x, y^ Means listed in the same row with different superscripts tend to be different (0.05 < *p* ≤ 0.10).

Figure 3 shows the effect of *Bacillus* probiotics on the mRNA expression of inflammatory genes in the ileum mucosa of weaned piglets. Compared with the CTR group, the BS1 + BS2 group significantly decreased *IL-8* mRNA expression (*p* < 0.05), and the BS1, BS2, and BS1 + BS2 groups significantly reduced TNF-α mRNA expression (*p* < 0.05). In addition, compared with the CTR group, the BS1 group showed a significant increase in *ZO-1* mRNA expression (*p* < 0.05). The addition of Bacillus to the diet had no significant effect on the expression of IL-6 mRNA, IL-10 mRNA and Occludin mRNA in the ileum mucosa of weaned piglets (*p* > 0.05).

### 3.10. mRNA Expression of Inflammatory Genes in Jejunum Mucosa

**Figure 4:**
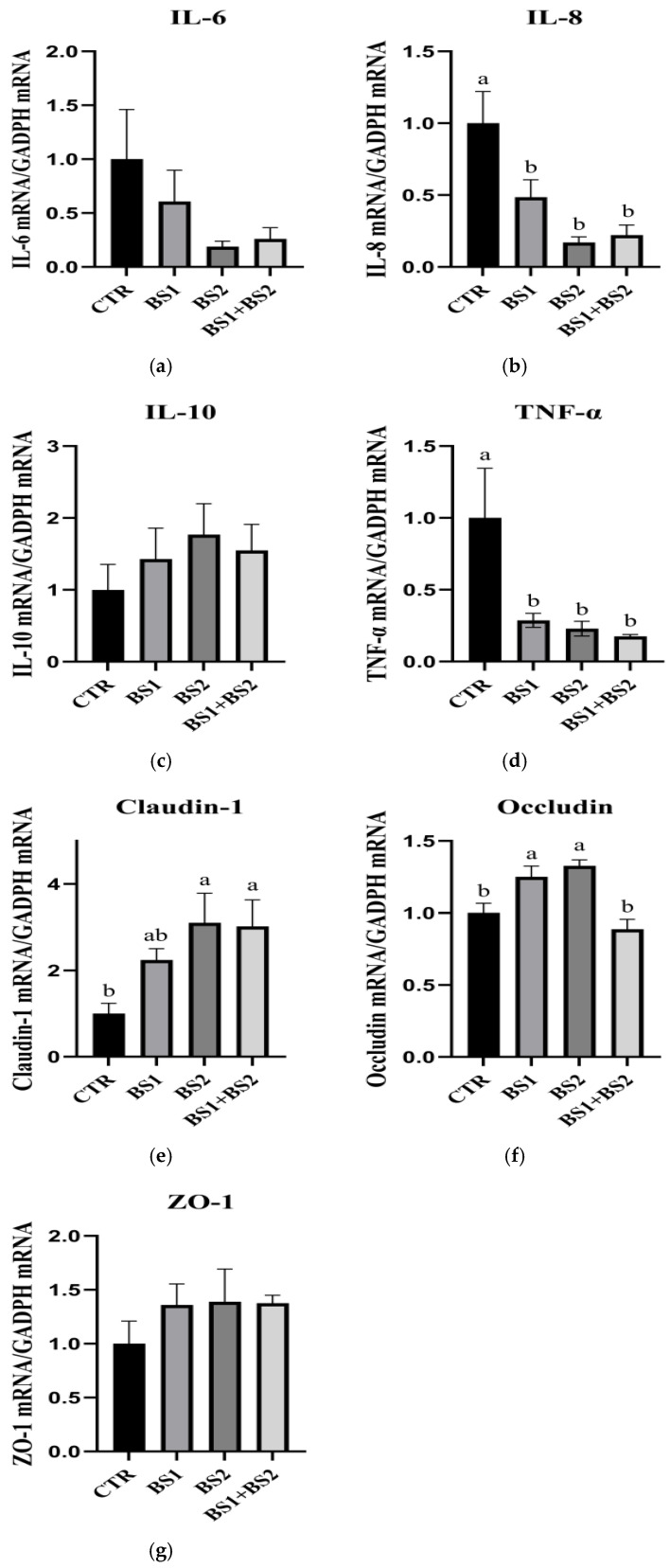
Effects of dietary *Bacillus* probiotics on mRNA expression of inflammatory genes in jejunum mucosa of weaned piglets. *IL-6*, interleukin-6; *IL-8*, interleukin-8; *IL-10*, interleukin-10; *TNF-α*, tumor necrosis factor-α. CTR, base diet without additives; BS1, CTR + 0.05% *Bacillus subtilis*; BS2, CTR + 0.05% *Bacillus pumilus*; BS1 + BS2, CTR + 0.05% *Bacillus subtilis* + 0.05% *Bacillus pumilus*. (**a**) the expression of IL-6 mRNA; (**b**) the expression of IL-8 mRNA; (**c**) the expression of IL-10 mRNA; (**d**) the expression of *TNF-α*; (**e**) the expression of Claudin-1 mRNA; (**f**) the expression of Occludin mRNA; (**g**) the expression of ZO-1 mRNA. n = 8. ^a, b^ Means with different superscripts means a significant difference (*p* < 0.05).

Figure 4 shows the effect of *Bacillus* probiotics on the mRNA expression of inflammatory genes in the jejunum mucosa of weaned piglets. Compared with the CTR group, the BS1, BS2 and BS1 + BS2 groups significantly decreased *IL-8* mRNA (*p* < 0.05) and considerably reduced *TNF-α* mRNA expression (*p* < 0.05). In addition, compared with the CTR group, the BS2 and BS1 + BS2 groups significantly increased *Claudin-1* mRNA expression (*p* < 0.05). Compared with the CTR and BS1 + BS2 groups, the BS1 and BS2 groups significantly increased the expression of *Occludin* mRNA (*p* < 0.05). The addition of *Bacillus* in the diet had no significant effect on the expression of *IL-6* mRNA, *IL-10* mRNA and *ZO-1* mRNA in the jejunum mucosa of weaned piglets (*p* > 0.05).

## 4. Discussion

The use of probiotics in pig production is increasingly favored because it allows avoidance or partially replacing antibiotics in feed, reducing post-weaning diarrhea and maintaining gastrointestinal health, and ultimately improving the growth performance of piglets. A large number of studies have shown the beneficial effects of probiotics in alleviating weaning stress and reducing diarrhea [20,21,22]; the results of this study showed that the combined addition of the BS1 + BS2 group could significantly increase the ADG and ADFI of days 0–14 and considerably reduce the diarrhea rate; at the same time, we observed that the BS2 group had higher ADG and ADFI on days 0–42, and the addition of *Bacillus* could significantly reduce the diarrhea rate on days 0–42. Other researchers have found some different results. In terms of BW, Menegat et al. [23] found that there was no direct evidence that there was a difference between the piglet diet supplemented with commercial probiotic products (calcosporin: *Bacillus subtilis* C-3102) and the piglet diet without commercial probiotic products. The different conclusions may be due to differences in dietary composition or interactions with dietary additives [24]. In addition, we observed that the ADG and ADFI of the BS1 group were slightly higher than those of the BS2 and BS1 + BS2 groups on days 28–42, but its FCR was lower than that of the BS2 group on days 28–42. This may indicate that piglets in the BS1 group may be in a more active growth state. The reason for this phenomenon may be the different growth-promoting mechanisms of probiotics. The BS1 group may improve growth performance by optimizing the intestinal environment for digestion. In contrast, the BS2 group and the BS1 + BS2 group are more focused on repairing the intestinal barrier and reducing inflammatory loss to enhance nutrient absorption efficiency [5]. *Bacillus* can produce a variety of digestive enzymes in the intestinal tracts of animals, such as proteases, lipases, and amylases [25]. At the same time, *Bacillus* produces amino acids, growth factors and other nutrients in the process of intestinal colonization and growth, which promotes metabolism in animals [26]. Improving nutrient apparent digestibility is a key factor for improving growth performance. Digestive enzymes and nutrients produced by *Bacillus* in the intestine may have a certain effect on improving the digestibility of piglets and thus affect growth performance. In our study, dietary supplementation with *Bacillus* probiotics improved nutrient digestibility, growth performance, and fecal consistency, which is consistent with those of Hu et al. [22]. Wu et al. [27] reported that the addition of fructooligosaccharides and *Bacillus licheniformis* alone or in combination could significantly improve the digestibility of CP and P, which was consistent with the results of this study.

Weaning stress is usually caused by different physical environments, exposure to pathogens, and changes in diet [28]. The primary antioxidant mechanism is a system composed of antioxidant enzymes and biological antioxidants, which synergistically maintain the generation and scavenging of free radicals, including SOD, GSH-Px [29]. SOD can catalyze the conversion of harmful superoxide to hydrogen peroxide and water, so an increase in SOD activity indicates enhanced antioxidant capacity. MDA is a metabolite of lipid peroxidation and a biomarker of oxidative stress. In this experiment, we observed that piglets in the BS1 + BS2 group had higher SOD activity and lower MDA levels, consistent with the study by Wang et al. [30]. At the same time, the antioxidant enzymes secreted by intestinal epithelial cells are the first line of defense against intestinal redox imbalance [31]. The activity of antioxidant enzymes determines intestinal redox status, the expression of related genes, and the products of oxidative damage [32]. Studies have shown that adding Bacillus probiotics to the diet can effectively reduce MDA levels in jejunal mucosa and improve intestinal antioxidant capacity [33]. The change in antioxidant capacity mainly arises from two sources: endogenous synthesis and exogenous supplementation. The intestinal tract of piglets can absorb substances with antioxidant capacity, such as α-tocopherol and vitamin C, which are absorbed from the feed into the blood, thereby directly improving the serum antioxidant capacity [34,35]. At the same time, the antioxidant components in the serum can also be transported to the intestine via the blood to help supplement dietary intake and resist oxidative damage. This study found that adding Bacillus probiotics to the diet can improve antioxidant capacity in the serum and jejunal mucosa of piglets, similar to the results of Wu et al. [36]. This shows that *Bacillus* has a specific effect on maintaining the redox homeostasis of the piglet intestine.

Oxidative stress can trigger an inflammatory response, which directly aggravates the redox imbalance [37], and this response is closely associated with the levels of pro-inflammatory cytokines in the body [38]. According to the study, TNF-α concentration is considered an indicator of weaning stress, reflecting the physiological immune status of piglets during weaning [39]. Many studies have shown that *Bacillus subtilis* affects the concentration of serum inflammatory cytokines in piglets [40,41,42]. However, there are few studies on the effect of *Bacillus pumilus* on serum inflammatory cytokines in piglets. This study showed that compared with the CTR group, piglets supplemented with the BS1 and BS2 groups had lower TNF-α concentration on day 21, and piglets supplemented with *Bacillus* had lower TNF-α concentration on day 42. In addition, we noted that dietary supplementation with BS1, BS2, and BS1 + BS2 did not significantly affect the serum levels of IgA, IgG, or IgM in weaned piglets. This result indicates that, under experimental conditions, Bacillus did not induce a systemic humoral immune response [5]. Instead, their immunomodulatory effects are primarily exerted through regulating mucosal immunity and innate immunity, consistent with the observed improvements in intestinal barrier function and inflammatory factors.

The morphological structure of intestinal villi and crypts directly determines nutrient digestion and absorption, as well as the normal function of the intestinal mucosal barrier [43]. Changes in intestinal morphology, such as intestinal villus atrophy and crypt hyperplasia, can destroy intestinal mucosal barrier function and digestion and absorption capacity [44,45]. At the same time, weaning stress can also disrupt intestinal secretion of digestive enzymes, leading to diarrhea in piglets and thus affecting growth performance [46]. This experiment showed that piglets supplemented with BS2 or BS1 + BS2 had lower crypt depth in the ileum and jejunum, suggesting reduced inflammatory stimulation. Generally, the ratio of villus height to crypt depth affects intestinal morphology, which, in turn, influences nutrient digestion [47,48]. This experiment showed that the jejunum of piglets supplemented with BS2 and BS1 + BS2 had higher V:C, indicating improved nutrient digestion, as reflected in improved growth performance and nutrient apparent digestibility.

The intestinal mucosal epithelial barrier prevents the invasion of pathogenic microorganisms and toxic substances [49]. Tight junctions are an essential part of the intestinal mucosal epithelial barrier [50]. The destruction of tight junctions or their loss of function will increase intestinal permeability, allowing infection and inflammatory factors to enter the systemic circulation and ultimately leading to tissue damage and changes in tight junction proteins [51], namely claudin-1, Occludins, and ZO-1, which can lead to intestinal mucosal epithelial barrier dysfunction [52]. The abundance of intestinal functional genes plays a regulatory role in maintaining the integrity of the intestinal barrier [16]. The study of Cao et al. (2018) found that weaning stress significantly down-regulated the expression of tight junction protein genes (Occludin, Claudin-1) in the intestinal tract of piglets, while up-regulated oxidative stress genes (GPX2, SOD3); changes in the expression of these genes directly lead to impaired mitochondrial function in intestinal epithelial cells, which in turn destroys the integrity of the intestinal mucosal barrier and increases intestinal permeability [32]. The results in this experiment showed that compared with the CTR group, the BS1 group increased the expression level of *ZO-1* in the ileum of piglets and increased the expression level of Occludin in the jejunum; moreover, the BS2 group increased the expression level of *Claudin-1* mRNA in the jejunum of piglets. At the same time, the expression levels of *IL-8* and *TNF-α* in the ileum and jejunum were decreased, which was similar to the results of Zhang et al. [53]. These results suggest that supplementation of BS1 and BS2 increases the mRNA expression levels of the tight junction proteins mentioned above, which may be due to the fact that *Bacillus* competes with pathogens for binding sites on the intestinal epithelium and produces toxic compounds to pathogens that stimulate the immune system [54]. In general, the BS1 + BS2 group did not show a synergistic effect on many indicators, which may be due to the fact that the two probiotics did not form an optimal ratio.

## 5. Conclusions

The results of this experiment showed that the addition of *Bacillus subtilis* and *Bacillus pumilus* to the diet could improve the growth performance of piglets and reduce diarrhea incidence, improve the antioxidant capacity of serum and intestinal tract, reduce the influence of inflammatory cytokines on the body, and preserve a high degree of intestinal morphological integrity. Therefore, in the BS2 group, the nutrients and energy absorbed may be used more for growth than to counter bacterial invasion and inflammation. Future studies should incorporate metagenomic analyses to further explore the effects of *Bacillus subtilis* and *Bacillus pumilus* on the intestinal microbiota of piglets. Large-scale animal experiments are recommended to further evaluate the effects of the two *Bacillus* species on the growth performance of weaned piglets under commercial conditions.

## Data Availability

Data are available on request from the authors.

## Abbreviations

The following abbreviations are used in this manuscript:

EU	The European Union
BW	Body Weight
ADG	Average Daily Gain
ADFI	Average Daily Feed Intake
FCR	Feed Conversion Rate
ATTD	Apparent Total Tract Digestibility
AIA	Acid-insoluble Ash
DM	Dry Matte
CP	Crude Protein
Ash	Ash Content
Ca	Calcium
P	Phosphorus
EE	Ether Extract
GE	Gross Energy
SOD	Superoxide Dismutase
MDA	Malondialdehyde
GSH-Px	Glutathione Peroxidase
T-SOD	Total Superoxide Dismutase
CAT	Catalase
8-OHdG	8-hydroxydeoxyguanosine
T-AOC	Total Antioxidant Capacity
IL-6	Interleukin-6
IL-1β	Interleukin-1β
TNF-α	Tumor Necrosis Factor-α
IFN-γ	Interferon-γ
IgA	Immunoglobulin A
IgG	Immunoglobulin G
IgM	Immunoglobulin M
VH	Villus Height
CD	Crypt Depth
V:C	Villus Height To Crypt Depth Ratio
IL-8	Interleukin-8
IL-10	Interleukin-10
AST	Aspartate Aminotransferase
ALT	Alanine Aminotransferase
ALP	Alkaline Phosphatase
TP	Total Protein
ALB	Albumin
TG	Triglyceride
CHO	Cholesterol
HDL	High-Density Lipoprotein Cholesterol
LDL	Low-Density Lipoprotein Cholesterol
GLU	Glucose
